# Correction of Class I Bimaxillary Protrusion Using Sliding Mechanics With Mini-implant Anchorage: A Case Report

**DOI:** 10.7759/cureus.73973

**Published:** 2024-11-19

**Authors:** Abhita Malhotra, Rajat Mangla, Puneet Batra, Ashish K Singh

**Affiliations:** 1 Department of Orthodontics and Dentofacial Orthopaedics, Manav Rachna Dental College, Manav Rachna International Institute of Research and Studies, Faridabad, IND; 2 Department of Orthodontics and Dentofacial Orthopaedics, Maharishi Markandeshwar College of Dental Sciences and Research (MMCDSR), Maharishi Markandeshwar (Deemed to be University) (MMDU), Ambala, IND

**Keywords:** anchorage, bimaxillary protrusion, en masse, mini-implants, proclination

## Abstract

Class I bimaxillary protrusion is characterized by proclined incisors, a convex facial profile, procumbent lips, and increased lip strain. Treatment includes the extraction of premolars and the mesial movement of the proclined anterior teeth in the extraction spaces to correct the inclination. This case report describes the treatment of an 18-year-old male patient who presented with class I bimaxillary protrusion and procumbent lips. Clinical examination revealed a convex profile, potentially competent lips, and increased lip strain. The treatment plan included therapeutic extraction of all first premolars and complete retraction of the anterior teeth in the extraction spaces. Absolute anchorage was required, and therefore, mini-implants were placed in both the upper and lower arches. Sliding mechanics were used to retract the anterior teeth in the extraction spaces. The use of mini-implants for absolute anchorage allowed for complete retraction of the anterior teeth without the inadvertent mesial movement of the posterior teeth. Posttreatment, the inclination of the anterior teeth was corrected, achieving normal overjet and overbite. Lip procumbancy was reduced, resulting in a more harmonious facial profile.

## Introduction

Class I bimaxillary protrusion refers to a condition in which both the upper and lower jaw bases are prognathic or positioned forward relative to the cranial base. It is commonly seen across various ethnic populations, with a prevalence of 3.7%-68.8%, and is particularly prevalent in African and Asian populations [1-3]. The features of class I bimaxillary protrusion can vary among different ethnic populations. In Caucasian populations, it is often associated with a decreased interincisal angle and a skeletal pattern tending toward class II [4]. In African populations, both skeletal and dental protrusions are common, with an increased ANB angle [5]. South Asian populations show bidental protrusion over a normal skeletal base, along with an increased nasolabial angle and greater facial convexity [6]. Therefore, treatment must be planned according to the specific skeletal and dental parameters seen in that particular population.

In the literature, the term "bimaxillary protrusion" may be used to suggest bidental protrusion, wherein proclined upper and lower anterior teeth are positioned on orthognathic jaw bases. Treatment of class I bidental protrusion usually entails the extraction of all first premolars and retraction of the upper and lower anterior teeth. To achieve optimal aesthetic results, mesial movement of the posterior teeth must be prevented, and extraction spaces must be closed with complete retraction of the anterior teeth. Therefore, maximum or absolute anchorage may be required to achieve the best results [7]. 

Anchorage obtained from the second premolars and the first molars on either side is insufficient, leading to anchorage loss and mesial movement of the posterior teeth. To augment the anchorage, several methods have been employed, such as two-step retraction (first, retracting the individual canines followed by retraction of the four anterior teeth), incorporating the second molars into the anchorage unit, using headgear, or using transpalatal or Nance palatal arch. However, these methods may still result in some degree of anchorage loss [8,9]. 

Since the advent of mini-implants, challenging tooth movements have been attempted with greater confidence, without the risk of unwanted reactionary tooth movement. Their ease of placement and removal, minimal chairside time, and independence from patient compliance have made them increasingly popular among orthodontists. This case report describes the en masse retraction of the anterior teeth in a patient with class I bimaxillary dental protrusion, utilizing sliding mechanics and anchorage provided by mini-implants.

## Case presentation

An 18-year-old male patient presented to the Department of Orthodontics and Dentofacial Orthopedics with a chief complaint of forwardly positioned upper and lower anterior teeth. Clinical examination revealed a mesoprosopic facial form and a convex facial profile, as shown in Figure 1. The patient exhibited procumbent lips, an acute nasolabial angle, and a deep mentolabial sulcus.

**Figure 1:**
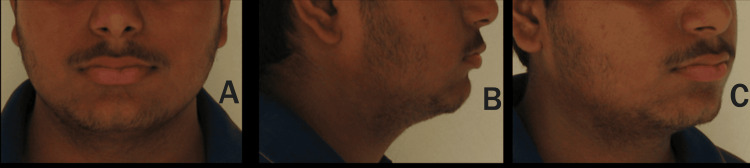
(A-C) Extraoral photographs of the patient

On intraoral examination, a class I molar relationship and a class I canine relationship were seen on the right and left sides. Both the upper and lower anterior teeth were proclined (Figure 2).

**Figure 2:**
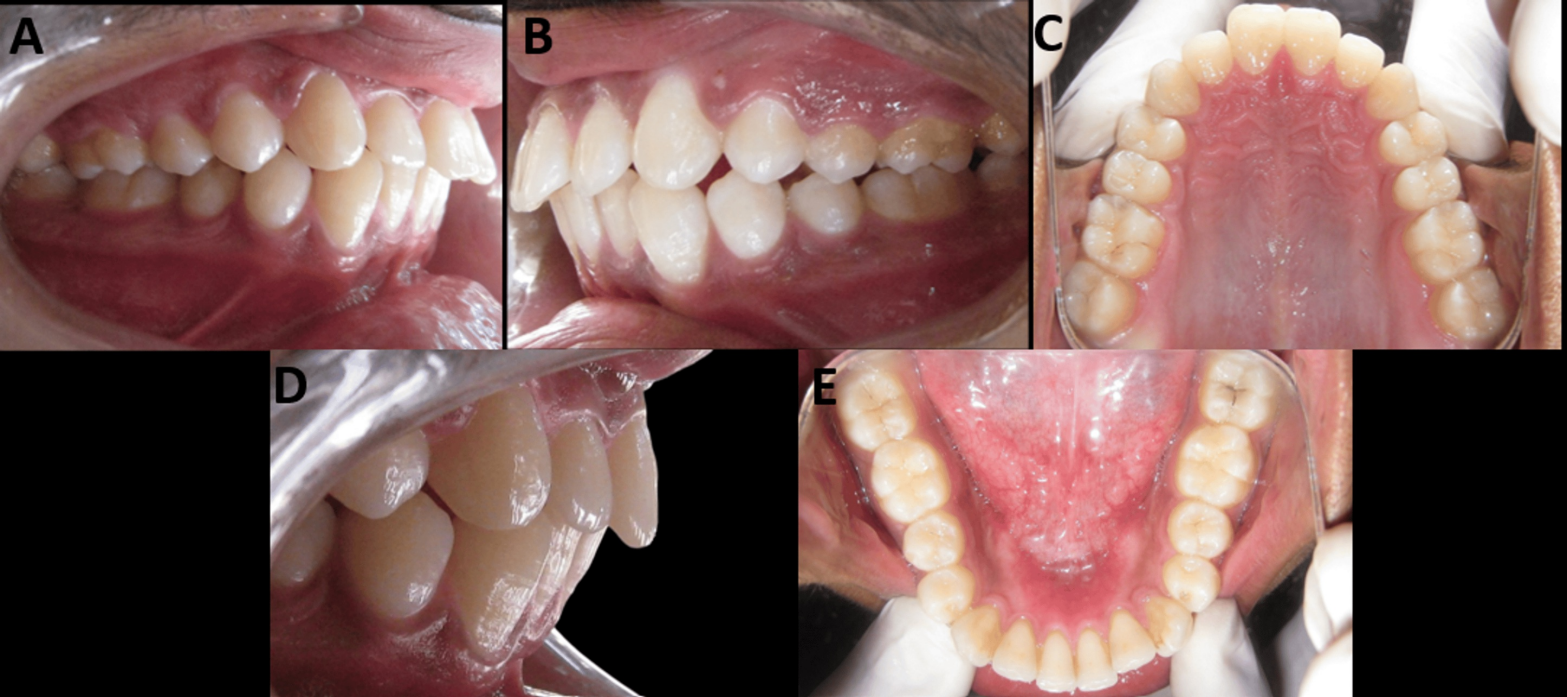
(A-E) Pretreatment intraoral photographs of the patient

Cephalometric examination (Figure 3A, Table 1) revealed a prognathic maxilla and mandible, with SNA = 89° and SNB = 84°. The skeletal pattern was class I (ANB = 5°, beta angle = 30°, yen angle = 120°), with a horizontal growth pattern (Frankfort mandibular plane angle (FMA) = 18°, Jarabak ratio = 73%) and a stage 6 cervical vertebral maturation index (CVMI). The upper incisor to NA angle was 34°/10 mm, and the lower incisor to NB angle was 35°/9 mm, suggesting proclination of the anterior teeth. The orthopantomogram showed the presence of all teeth, including the third molars, with no bone loss and good bone quality (Figure 3B). 

**Figure 3:**
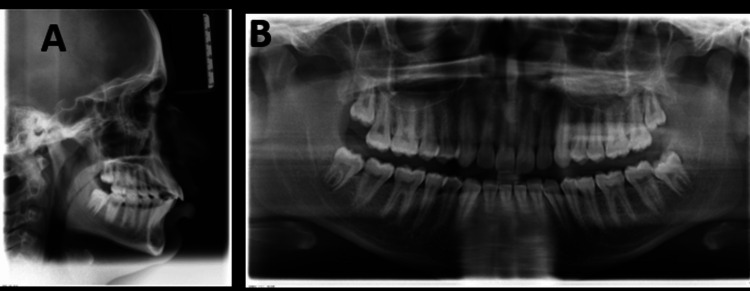
Pretreatment (A) cephalometric radiograph and (B) orthopantomogram

**Table 1:** Pretreatment cephalometric values SNA: sella-nasion-point A angle, SNB: sella-nasion-point B angle, ANB: point A-nasion-point B angle, FMA: Frankfort mandibular plane angle, UI: upper incisor, LI: lower incisor, SN: sella-nasion, PP: palatal plane, IMPA: incisor mandibular plane angle.

Parameters	Pretreatment value
SNA angle	89°
SNB angle	84°
ANB angle	5°
Beta angle	30°
Yen angle	120°
Jarabak ratio	73°
FMA	18°
UP-NA	36°/10 mm
LI-NB	35°/9 mm
LI to A-Pog	7 mm
IMPA	110°
Upper lip to E-line	+1 mm
Lower lip to E-line	+3.5 mm
Nasolabial angle	82°

For treatment, the extraction of all first premolars was decided, along with the utilization of the extraction spaces to correct the inclination of the anterior teeth. A 0.022” MBT appliance (3M Oral Care, Minnesota, USA) was chosen, and sliding mechanics were to be used for retraction. Since complete space closure by retraction of the anterior teeth was required and friction mechanics were to be used, absolute anchorage with mini-implant was planned in all four quadrants.

Orthodontic phase

Orthodontic treatment was started with a straight-wire appliance, and the arches were leveled and aligned with a 0.016” nickel-titanium (NiTi) wire, followed by a 0.018” stainless steel archwire, a 0.017 x 0.025” stainless steel archwire, and finally a 0.019 x 0.025” stainless steel archwire. The 0.019 x 0.025” stainless steel archwire was engaged for at least one month to allow for the expression of the tip and torque, ensuring passive engagement of the wire in the bracket slots, which was necessary for the use of sliding mechanics for retraction. 

For precise implant placement, an implant placement jig was made with a 0.017 x 0.025” stainless steel wire, as shown in Figure 4. This was engaged to the brackets on the second premolar and first molar. An intraoral periapical (IOPA) radiograph was taken soon afterward to ensure that the jig guided the implant placement in the interradicular region of the alveolar bone (Figure 5A). The template was made to ensure the highest placement of the implant in the alveolar bone in the region of the attached gingiva. This was done to prevent the deepening of the bite when retraction forces were applied from the mini-implant.

**Figure 4:**
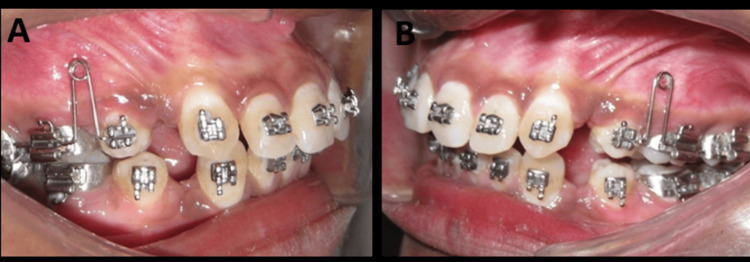
(A, B) Implant placement jig engaged in the premolar and molar bracket slots

**Figure 5:**
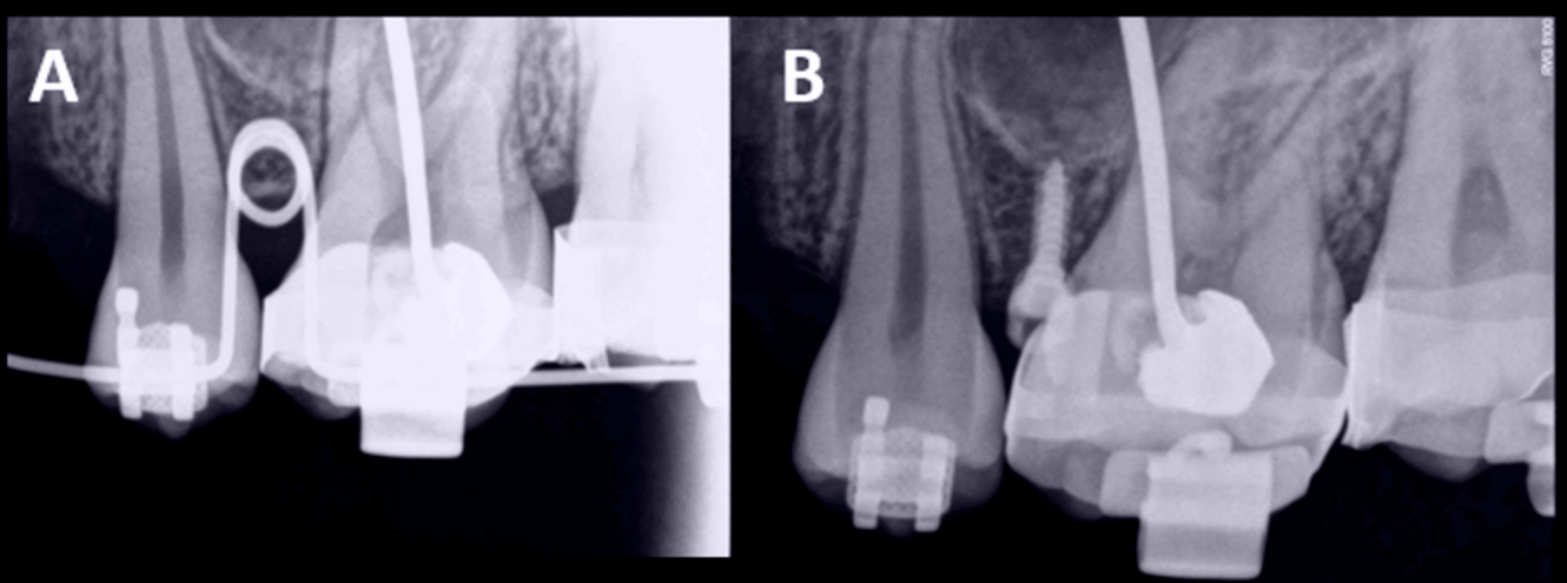
(A) IOPA radiograph to ensure interradicular position of the mini-implant placement jig. (B) IOPA radiograph after mini-implant placement in the interradicular bone between the second premolar and first molar IOPA: intraoral periapical.

Infiltration anesthesia (Lignox Indoco Warren, Indoco Pvt Ltd.; 2% lignocaine with 1:80,000 Adrenaline) was administered. Using an explorer, a bleeding point was made at the center of the jig to mark the site for implant insertion. The implant placement jig was then removed, and a 1.3 x 8 mm self-drilling mini-implant (S.K. Surgical Pvt. Ltd., Pune, India) was inserted at the marked site and checked for primary stability.

The IOPA X-ray was retaken to ensure the placement of the mini-implant in the interradicular region of the alveolar bone (Figure 5B). After one week, the implant was loaded with a retraction force of 150-200 g using a closed NiTi coil spring (G&H Orthodontics, Indiana, USA), calibrated with a Dontrix gauge (TP Orthodontics, Inc., Indiana, USA). Delayed loading of the implant is said to cause faster retraction of the teeth during the initial months of treatment [10]. Studies suggest that a delayed loading of 7-14 days can help reduce the risk of implant failure, as studies have reported displacement of the implant after loading [11].

Mini-implants were placed in all the four quadrants. The retraction mechanism consisted of three parts (Figure 6): (a) anterior segment, (b) posterior segment, and (c) retraction NiTi coil spring, with the force vector directed upward and posteriorly. The retraction coil spring was attached to a short power arm (5 mm) placed mesial to the canine bracket on the anterior segment and to the mini-implant on the posterior segment. This created an upward and posteriorly directed retraction force, preventing inadvertent bite deepening during the correction of the anterior teeth inclination and retraction.

**Figure 6:**
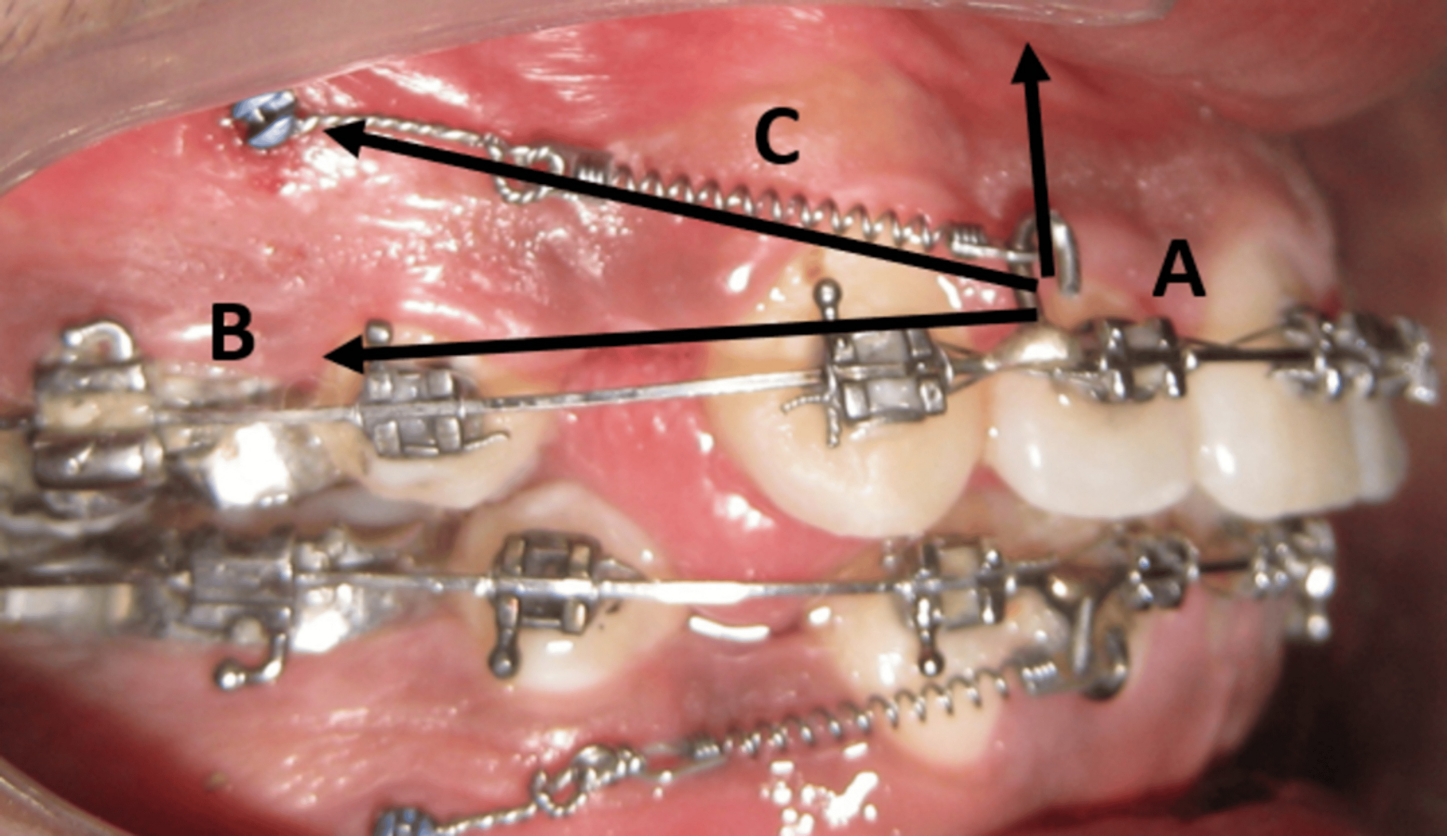
(A-C) Mini-implant is placed high in the alveolar bone. Note the force vector due to the highly placed implant

A segmental torque of 20° was added to improve the incisor inclination in the upper arch, close to the completion of space closure. A harmonious facial profile was achieved, and lip competency improved. There was also an improvement in the nasolabial angle and mentolabial sulcus. The correction of the anterior teeth inclination, along with a normal overjet and overbite, was noted without any loss of anchorage (Figure 7, Figure 8). The mini-implants were removed after space closure.

**Figure 7:**
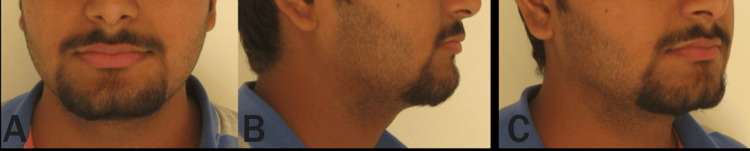
(A-C) Extraoral photographs of the patient after complete retraction of the anterior teeth

**Figure 8:**
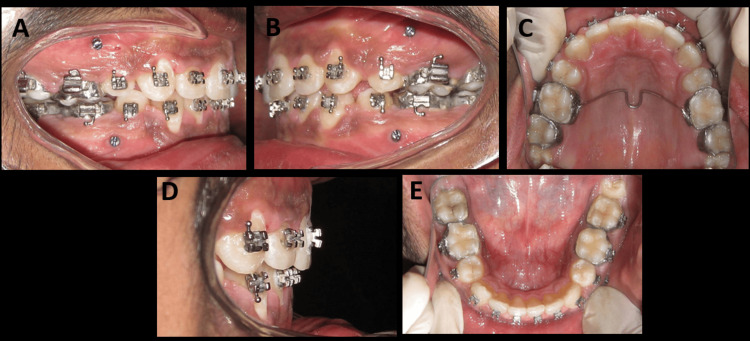
(A-E) Intraoral photographs after retraction of the anterior teeth

The cephalometric evaluation revealed a reduction in the SNA and SNB angles, a correction of the inclination, an increased nasolabial angle, and a reduced mentolabial sulcus. Lip procumbency decreased, and the facial profile improved, as seen in the cephalometric superimposition (Figure 9, Table 2).

**Figure 9:**
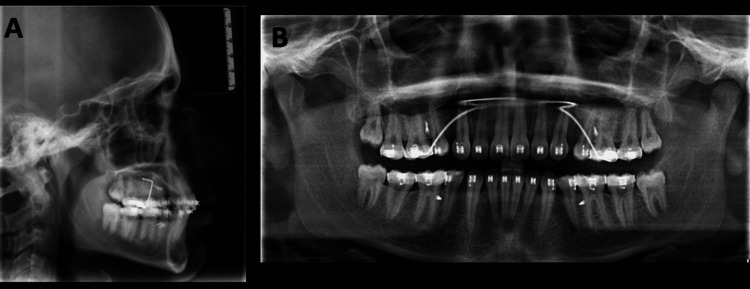
Post-retraction (A) cephalometric radiograph and (B) orthopantomogram

**Table 2:** Pretreatment and posttreatment cephalometric values SNA: sella-nasion-point A angle, SNB: sella-nasion-point B angle, ANB: point A-nasion-point B angle, FMA: Frankfort mandibular plane angle, UI: upper incisor, LI: lower incisor; SN: sella-nasion, PP: palatal plane, IMPA: incisor mandibular plane angle.

Parameters	Pretreatment value	Posttreatment
SNA	89°	84°
SNB	84°	83°
ANB	5°	2°
Jarabak ratio	73%	72%
UI-SN	121°	102°
UI-PP	123°	110°
UP-NA	30°/10 mm	15°/4 mm
LI-NB	33°/9 mm	18°/3 mm
LI-A-Pog	7 mm	0 mm
IMPA	110°	91°
Upper lip to E-line	+1 mm	-0.5 mm
Lower lip to E-line	+3.5 mm	-1 mm
Nasolabial angle	82°	95°
Mentolabial sulcus	9 mm	7 mm

The total active treatment duration was 19 months. Full-time retention was kept for 10 months, and then nighttime wear was suggested for another eight months to prevent relapse due to the opening of the extraction spaces. 

## Discussion

A class I bimaxillary protrusion case requires careful diagnosis of both the teeth and the jaws [3]. The underlying etiology must be assessed before formulating a treatment plan. Cases with forwardly positioned jaws relative to the cranial base may require orthodontic treatment or even a surgical correction to achieve an aesthetic result. In cases where class I bimaxillary protrusion is due to dentoalveolar protrusion, orthodontic therapy alone may be sufficient. Since the anterior teeth are proclined on normal jaw bases, extraction of the first premolars is performed, and the anterior teeth are moved distally into the extraction spaces. To achieve complete retraction of the anterior teeth in the premolar extraction spaces, careful planning of the biomechanics is required [4].

Class I bidental protrusion is typically seen in the Indian population. The characteristics include proclined teeth in both jaws, increased lip strain, and a convex facial profile [4]. In the current case report, class I bimaxillary dental protrusion exhibited proclined teeth, protruded lips, and a convex profile, resulting in an unaesthetic appearance for the patient. It was decided to extract all four premolars and utilize the extraction spaces fully to correct the inclination of the anterior teeth. This approach was aimed at reducing facial convexity and improving lip competence. Loss of anchorage due to mesial movement of the posterior teeth would have compromised the amount of anterior teeth retraction. Therefore, biomechanics had to be carefully planned, and the reinforcement of the anchorage unit was required.

Before mini-implants became available to orthodontists, several methods were employed to reinforce anchorage. The two-step retraction, where individual canine retraction was done first, followed by retraction of the anterior teeth, was a common technique. However, this method involved longer treatment durations than en masse retraction of the teeth, and therefore, many clinicians preferred en masse retraction to shorten the treatment time [12]. In other cases, second molars were included in the anchorage unit to prevent mesial movement during the retraction of the anterior teeth. Transpalatal or Nance palatal arch may also be used. However, studies reveal that this could also lead to some loss of anchorage, compromising the results [9,13,14].

Mini-implants, unlike conventional anchorage methods, do not involve the mesial movement of the posterior teeth. Since they are anchored within the alveolar bone, direct anchorage can be taken from them, preventing mesial tipping of the anchorage unit [15].

Mini-implants also provide biomechanical advantages. The placement of the mini-implant posteriorly and the length of the power arm positioned anteriorly can be customized to meet specific requirements and needs. Strategic placement of implants, high in the alveolar bone, provides the biomechanical advantage of preventing bite deepening during retraction. This allows the retraction force to be accompanied by an intrusion force vector, preventing the extrusion of the anterior teeth during space closure [16]. The orthodontist checks the retraction force, and activation is done only when the force levels are below the optimal force required for retraction, thereby reducing chairside time and providing an efficient and comfortable orthodontic treatment for the patient.

A short power arm was placed between the lateral incisor and canine bracket. As suggested by Upadhyay et al. [17], a 5-mm power arm mesial to the canine bracket was utilized, which was occlusal to the center of resistance of the anterior teeth. The NiTi coil spring spanned from the short power arm to the mini-implant placed higher in the alveolar bone. This provided backward and upward force vectors, leading to both intrusion and retraction of the anterior teeth. The retraction force was applied below the center of resistance of the anterior teeth, creating a clockwise moment that would cause deepening of the bite. However, the intrusive component of the force counteracts this clockwise moment, causing controlled tipping of the anterior teeth. Also, the retraction force applied to the power arm was calibrated to 150 g using a Dontix gauge. Therefore, the moment of force generated was not high, preventing excessive mesial tipping or torque loss of the anterior teeth. Since controlled tipping was required for correction of the inclination, the power arms were not kept long because longer arms would have caused the retraction force to be more horizontal and closer to the center of resistance of the anterior teeth, causing greater translation instead of tipping.

A stiff stainless steel wire with dimensions of 0.019 x 0.025” was used. According to Tominaga et al. [18], when a wire of lesser dimension is used in the brackets, causing greater play of the wire within the bracket slot, greater uncontrolled tipping will ensue. This will cause labial movement of the roots of the anterior teeth during retraction. Therefore, a heavier stainless steel archwire, such as a 0.019 x 0.025” stainless steel, is recommended for en masse retraction with sliding mechanics to prevent archwire deflection during retraction.

In our case report, we leveraged the biomechanical advantage of higher mini-implant placement for space closure. The utilization of mini-implants for retraction helped provide absolute anchorage, allowing for complete retraction of the anterior teeth. The force vector prevented the deepening of the bite during space closure. Complete retraction of the anterior teeth improved the profile of the patient, reduced lip strain, and resulted in lip competence. Ultimately, this led to a more harmonious facial profile.

## Conclusions

This case report describes the treatment of a patient with class I bimaxillary dental protrusion, utilizing mini-implants for anchorage following the extraction of all first premolars.

Loss of anchorage, if any, may compromise the aesthetic outcomes. Since the patient showed protruded lips and proclined anterior teeth, the case was treated by en masse retraction of the anterior teeth in the extraction spaces. Absolute anchorage was required, and therefore, mini-implants were chosen to be placed in all four quadrants to support the retraction mechanics. The application of retraction forces from the mini-implants resulted in complete retraction of the anterior teeth and prevention of bite deepening during the process. This resulted in a reduction in lip strain and improved facial aesthetics.
